# Fluoroquinolone Exposure and Other Factors Associated with Achilles Tendon Rupture: A Nationwide Nested Case–Control Study

**DOI:** 10.3390/jcm15218404

**Published:** 2026-10-28

**Authors:** Jae Yong Park, Hye Chang Rhim, Young-Min Kwon, Jihyun Hwang, Collin Lee, Yagop Shin, Sung Jae Kim

**Affiliations:** 1 Department of Orthopedic Surgery, Hallym University Sacred Heart Hospital, Hallym University College of Medicine, Anyang 14068, Republic of Korea; getfours@gmail.com; 2 Department of Physical Medicine and Rehabilitation, Harvard Medical School, Spaulding Rehabilitation Hospital, Boston, MA 02115, USA; hrhim@mgh.harvard.edu; 3 Foot & Ankle Research and Innovation Lab (FARIL), Department of Orthopaedic Surgery, Harvard Medical School, Massachusetts General Hospital, Boston, MA 02115, USA; 4 Department of Orthopedic Surgery, Hallym University Dongtan Sacred Heart Hospital, Hwaseong 18450, Republic of Korea; kwonym0814@gmail.com; 5 The George Washington University School of Medicine and Health Sciences, Washington, DC 20037, USA; jihyun.hwang@gwu.edu; 6 Department of Biology, University of Maryland–College Park, College Park, MD 20742, USA; cjlee2022@gmail.com; 7 Department of Orthopedic Surgery, Hanyang University Seoul Hospital, Seoul 04763, Republic of Korea; jacob960503@gmail.com; 8 Department of Orthopedic Surgery, Hanyang University Guri Hospital, Guri 11923, Republic of Korea

**Keywords:** fluoroquinolone, levofloxacin, Achilles tendon rupture

## Abstract

**Background/Objectives**: Fluoroquinolones have been associated with tendon disorders, including Achilles tendon rupture (ATR). We investigated the associations of individual fluoroquinolone prescriptions and patient characteristics with ATR in a nationwide Korean claims dataset. **Methods**: This nested case–control study used Korean National Health Insurance Service data from 2009 to 2019. We included 40,240 ATR cases identified by KCD code S86.0 and 160,960 risk-set-sampled controls. Exposure was defined as a fluoroquinolone prescription lasting more than six days within 180 days before the index date. Poisson regression described population rates; unconditional multivariable logistic regression estimated adjusted odds ratios (aORs). **Results**: The overall incidence of ATR was 29.1 per 100,000 persons and increased during the study period. Levofloxacin prescriptions were associated with higher odds of ATR (aOR 1.28, 95% CI 1.13–1.44). In age-stratified analyses, the levofloxacin estimate was 1.08 (95% CI 0.82–1.41), 1.25 (1.07–1.46), and 1.66 (1.23–2.23) in those aged 23–39, 40–64, and ≥65 years, respectively; interaction was not formally tested. Higher BMI (30–34.9 kg/m^2^: aOR 2.20, 95% CI 2.09–2.32) and other patient characteristics were also associated with ATR in the overall sample. **Conclusions**: Levofloxacin exposure was associated with ATR, particularly in older adults, although outcome specificity, exposure classification, residual confounding, and incomplete accounting for matching limit causal interpretation. The data do not establish the comparative safety of other fluoroquinolones or fluoroquinolone-specific effects of patient characteristics.

## 1. Introduction

The Achilles tendon is one of the most frequently ruptured tendons in the human body [1]. Reported incidence varies by healthcare setting and ascertainment method. A US emergency department study reported 1.8 to 2.5 Achilles tendon ruptures per 100,000 person-years between 2012 and 2016 [2]; population-based studies in Ontario, Finland, and Sweden have reported different rates and increasing trends [3,4,5]. Physical activity among aging adults, sex- and age-related variation, seasonal patterns, obesity, and medication exposures have been associated with Achilles tendon injuries and rupture in previous studies [4,6,7,8,9,10,11,12].

Fluoroquinolones are broad-spectrum antibiotics with good oral bioavailability and tolerability that are widely used to treat urinary, respiratory, and gastrointestinal infections [13]. However, over the years, some serious adverse events such as severe hemolytic-uremic syndrome, hepatotoxicity, QT prolongation, and dysglycemia have been reported and resulted in the market withdrawal of some of these antibiotics [14,15,16]. Cases of tendinopathy and tendon ruptures were also reported soon after their introduction to the market, and in 2008, the US Food and Drug Administration (FDA) added a “black box” warning citing an increased risk of tendon rupture to systemic fluoroquinolones [17]. More recently in 2018, the European Medicines Agency (EMA) also recommended restricting the use of fluoroquinolones after reviewing safety data on disabling and potentially long-lasting adverse events including tendon injuries [18].

Prior observational studies and regulatory assessments have linked fluoroquinolone treatment to tendon adverse events, but agent-specific estimates and clinically relevant exposure windows vary across studies [12,17,18]. Associations between patient characteristics and tendon injury in the overall population do not necessarily identify modifiers of fluoroquinolone-related effects. A nationwide claims-based analysis can describe these associations while requiring caution in causal interpretation.

Therefore, this study aimed to (1) describe the incidence of ATR in South Korea and (2) examine associations of individual fluoroquinolone prescriptions and selected patient characteristics with ATR in a nested case–control sample.

## 2. Materials and Methods

### 2.1. Data Source

The data used in this study were obtained from the Korean National Health Insurance Service (NHIS) which covers approximately 97% of the population in South Korea as a national insurance service. The database of NHIS consists of individual demographic data (including sex, age, residential area, socioeconomic variables), health insurance claim data (diagnostic codes, Korean Classification of Disease (KCD) and codes for clinical procedure) and general health examination data. In South Korea, general health examination is conducted every two years for the entire country for those who are 20 years old or above [19,20]. This study was approved by the Institutional Review Board of Dongtan Sacred Heart Hospital (IRB#HDT 2020-06-008) and performed in accordance with the Declaration of Helsinki. The informed consent of the participants was waived by the Institutional Review Board of Dongtan Sacred Heart Hospital because all data in this study were totally anonymized with the NHIS providing a de-identified dataset.

### 2.2. Study Design and Population

Achilles tendon rupture (ATR) was identified using Korean Classification of Diseases (KCD) code S86.0 (injury of Achilles tendon), rather than the broader S86 category; the same code has been used to identify ATR in previous Korean NHIS studies [21]. Because S86.0 also includes Achilles tendon injuries other than complete rupture, and cases were not confirmed by imaging, chart review, or Achilles tendon repair procedure codes, “ATR” hereafter denotes this claims-defined outcome. Records were initially identified between 2002 and 2019 in the NHIS database. Adults aged 23 years or older were eligible. Although the initial identification covered 2002–2019, the incidence analysis focused on 2009–2019 because earlier health examination information was insufficient. Of 122,161 identified cases in the incidence analysis, 81,594 were excluded because of unavailable health examination data and 327 because of missing analytic variables, leaving 40,240 cases for the nested case–control analysis.

**Figure 1:**
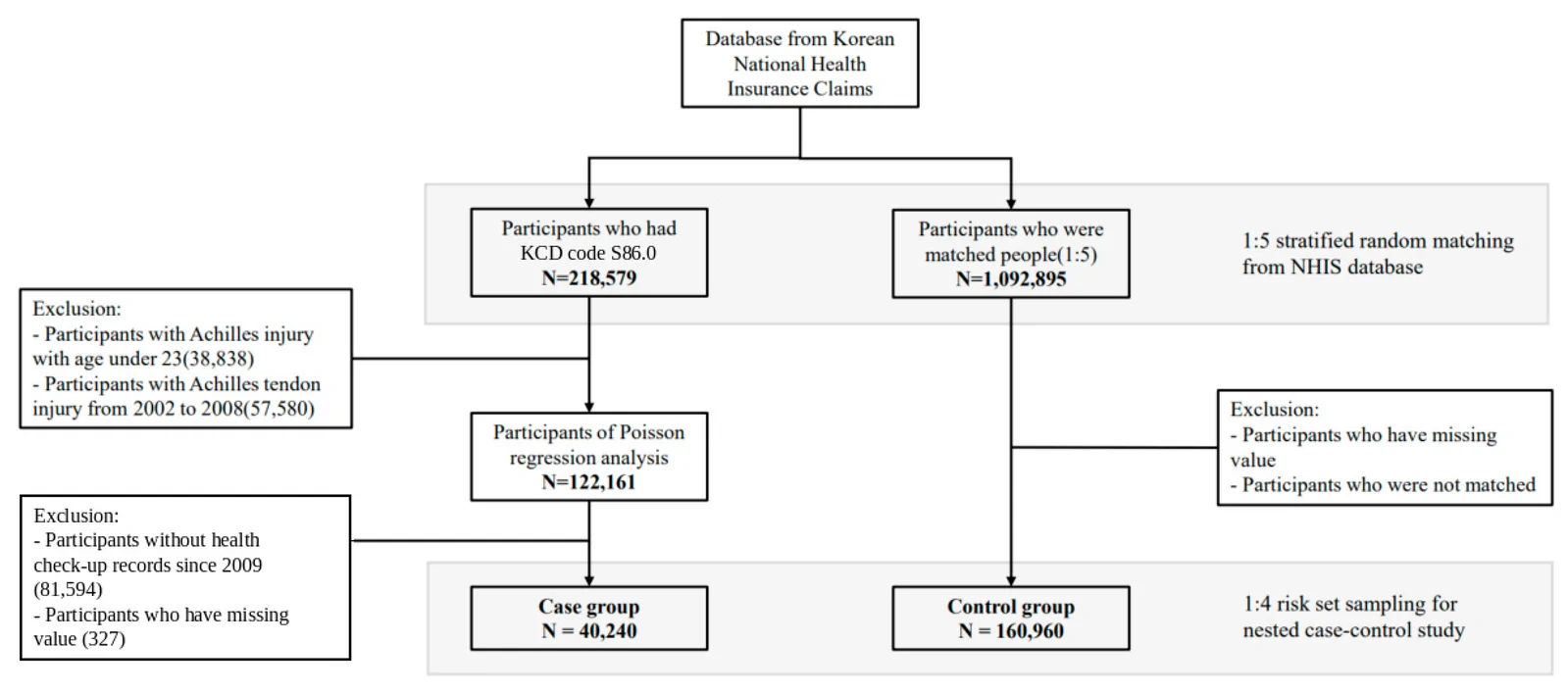
Study flow diagram showing initial 1:5 control preselection followed by final 1:4 risk-set sampling. The 122,161 cases included in the incidence analysis were reduced by exclusion of unavailable health examination data (*n* = 81,594) and other missing analytic values (*n* = 327), leaving 40,240 analytic cases and 160,960 control selections. Cases were identified by KCD code S86.0.

A sex- and age-stratified 1:5 sampling step first established a potential control pool. From this pool, four controls were selected per analytic case by risk-set sampling at the case index date, matched on sex, birth year (within ±2 years), and health examination year (within ±2 years), yielding 160,960 control selections. As risk-set sampling allows a person to be sampled at multiple risk-set dates or to become a later case, repeated selections need not represent erroneous duplicates. The extent of such repeated selection was not separately evaluated. The study flow is presented in Figure 1.

### 2.3. Definition of Fluoroquinolone Exposure

Fluoroquinolone exposure was operationally defined as a recorded oral or intravenous prescription lasting more than six days (at least seven days) within 180 days before the index date [22,23]. This analytic cutoff was not intended to imply a validated minimum duration of tendon toxicity. Tendon disorders have been reported after shorter courses, and exposure-related risk may be greatest during the first several weeks; thus, the broad lookback window and exclusion of shorter courses may affect exposure classification. The agents examined were ciprofloxacin, levofloxacin, fleroxacin, lomefloxacin, ofloxacin, norfloxacin, and moxifloxacin. Treatment duration, cumulative dose, prescription recency, and overlapping or repeated antibiotic courses were not separately modeled. Prescription records do not confirm medication ingestion.

### 2.4. Statistical Analysis

Poisson regression was used to describe annual ATR incidence and incidence rate ratios (IRRs) with 95% confidence intervals (CIs). Denominators came from the Korea National Statistical Office population estimates, stratified by sex, age, and year. Rates were reported per 100,000 persons. Independent variables were sex, age group, year, and month. Age categories were 23–39, 40–64, and ≥65 years. Because ascertainment was based on KCD S86.0, these estimates represent claims-defined ATR rather than independently validated complete rupture.

A nested case–control analysis used the first recorded ATR date during the study period as the case index date. Controls were sampled from individuals at risk at each corresponding index date. Under incidence-density sampling, individuals could be selected as controls at earlier index dates and could subsequently become cases; the resulting sample contains risk-set selections rather than necessarily unique persons.

Group characteristics were compared using independent-sample t-tests and chi-square tests for continuous and categorical variables, respectively. The original multivariable analysis used unconditional logistic regression, not matched-set conditional logistic regression, to model ATR. Covariates were sex, age group, residence, socioeconomic category, comorbidities, Charlson comorbidity index (CCI), body mass index (BMI), proteinuria, hemoglobin (g/dL), total cholesterol, aspartate aminotransferase (AST), alanine aminotransferase (ALT), creatinine, alcohol consumption, and physical activity score (PAS). PAS was calculated as (3a + 5b + 8c)/7, where a, b, and c denote reported weekly days of light-, moderate-, and vigorous-intensity activity, respectively [24]. Comorbid diagnoses were identified before the outcome index date but were not necessarily established to precede antibiotic exposure. Concomitant systemic corticosteroid exposure was not incorporated into the reported regression model. The unconditional model did not preserve the matched risk-set strata, which is an important constraint on interpreting the adjusted estimates, particularly those for matching variables.

Age-stratified estimates for ciprofloxacin, levofloxacin, and ofloxacin are presented in Supplementary Table S3; other agents had few exposed participants. Supplementary Table S4 provides alternative existing covariate-adjustment specifications rather than formal fluoroquinolone-by-covariate interaction tests. No interaction terms were formally evaluated in the reported results, and no multiplicity adjustment was applied to the agent-specific comparisons. Analyses using 30- or 90-day exposure windows, excluding Achilles tendinitis, restricting to completely unexposed reference participants, or additionally adjusting for corticosteroids, were not undertaken. The available results do not include a formal assessment of multicollinearity, fit, or calibration.

Statistical significance was set at *p* < 0.05. R software version 3.4.3 (The R Foundation for Statistical Computing, Vienna, Austria) and SAS Enterprise Guide version 7.15 (SAS Institute, Cary, NC, USA) were used in all analyses.

### 2.5. Use of Generative Artificial Intelligence

ChatGPT (GPT-6, OpenAI) was used to assist with English-language editing and with drafting revised wording of the text during manuscript revision. The tool was not used for study design, data extraction, statistical analysis, or interpretation of results. All AI-assisted text was reviewed, edited, and approved by the authors, who take full responsibility for the content of the manuscript.

## 3. Results

### 3.1. Incidence of ATR and Incidence Rate Ratios

**Figure 2:**
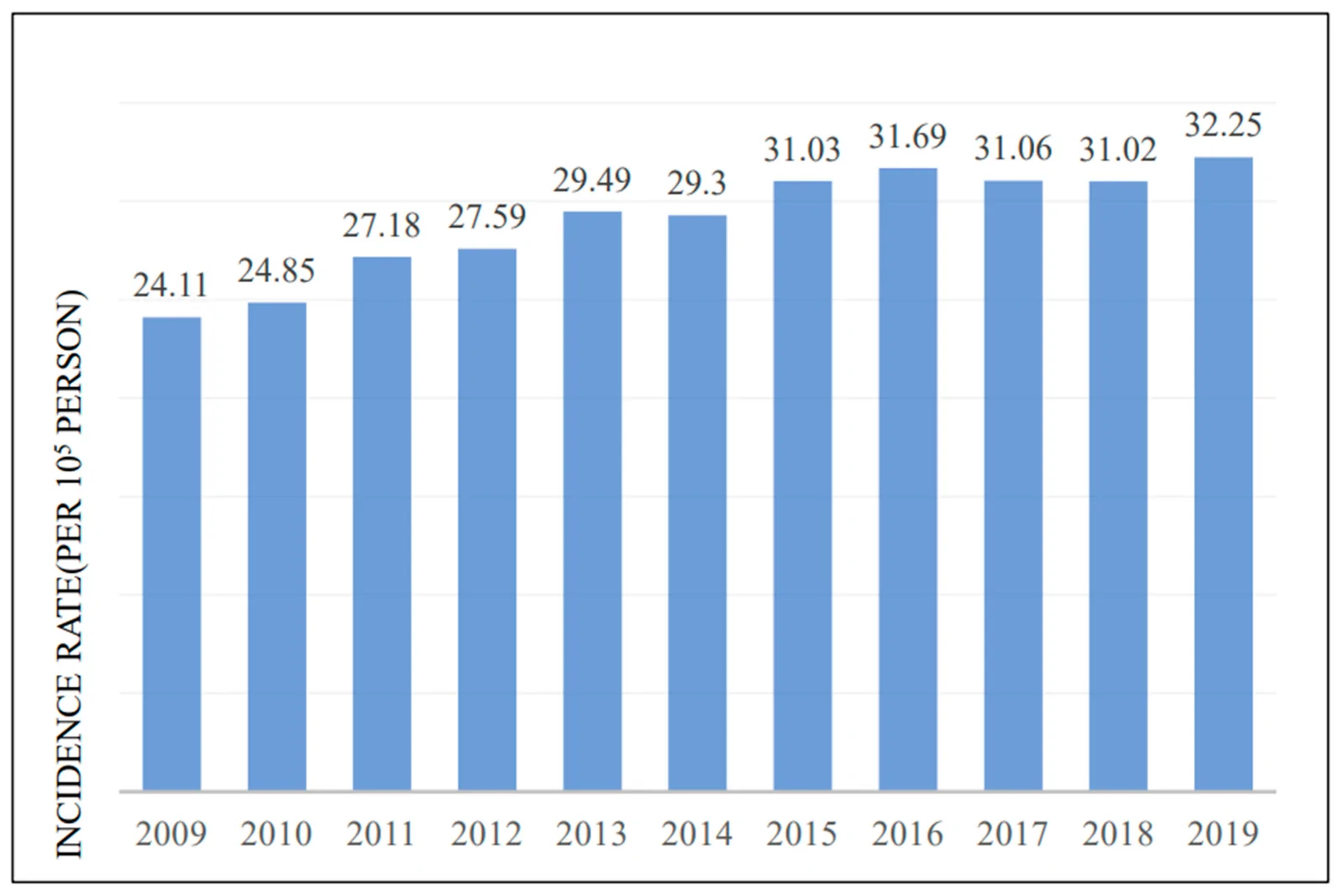
The annual incidence rate of Achilles tendon rupture in South Korea.

A total of 40,240 ATR cases and 160,960 control selections were included in the nested case–control analysis (Figure 1). The overall incidence of ATR from 2009 to 2019 was 29.1 per 100,000 persons: 40.7 per 100,000 persons in men and 17.1 per 100,000 persons in women; female sex was associated with lower population incidence (IRR 0.41, 95% CI 0.41–0.42). Adults aged ≥65 years had a lower incidence than adults aged 23–39 years (IRR 0.52, 95% CI 0.50–0.53). Annual incidence increased from 24.11 per 100,000 persons in 2009 to 32.25 in 2019 (Table 1; Figure 2). These are claims-defined rates, not imaging- or surgery-confirmed rupture rates.

**Figure 3:**
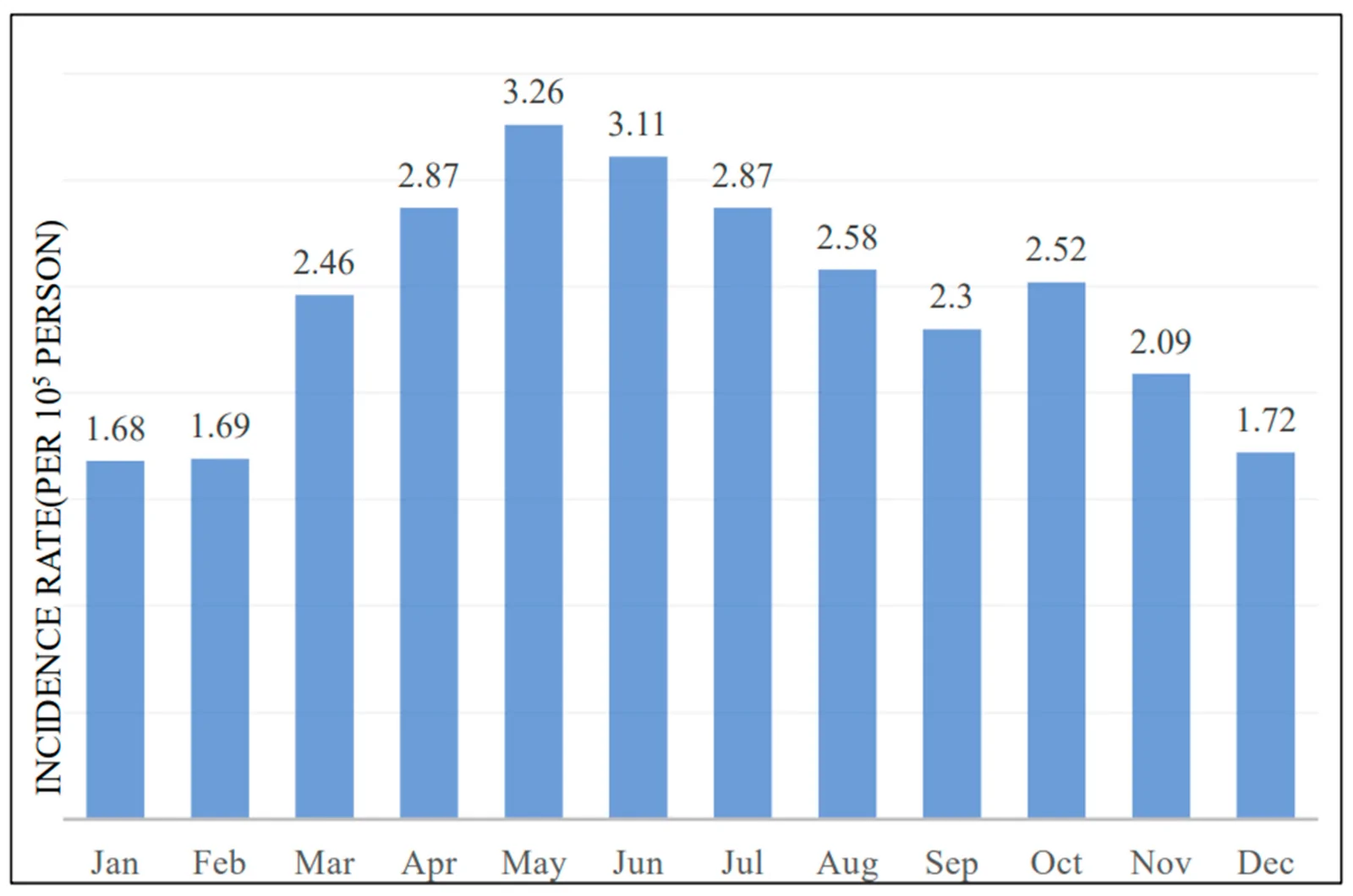
The monthly incidence rate of Achilles tendon rupture in South Korea from 2009 to 2019.

Figure 3 depicts the seasonal distribution of ATR. The reported rate increased from January (1.68 per 100,000 persons) to May (3.26 per 100,000 persons) and subsequently declined toward December (1.72 per 100,000 persons); the May-to-January IRR was 1.94 (95% CI 1.88–1.99).

### 3.2. Demographic Information and Univariate Analysis

The characteristics of the 40,240 case selections and 160,960 control selections are summarized in Table 2. Sex and age distributions were similar, consistent with the matching design. Achilles tendinitis and plantar fasciitis diagnoses were more frequent among cases (8.9% versus 0.5%, and 2.6% versus 0.7%, respectively; both *p* < 0.001). The proportion with recorded levofloxacin exposure was 1.0% among cases and 0.7% among controls (*p* < 0.001); for ciprofloxacin it was 0.8% and 0.7%, respectively (*p* = 0.036). These are unadjusted group comparisons.

### 3.3. Results from Multivariable Logistic Regression

Table 3 presents the previously estimated covariate-adjusted odds ratios from unconditional logistic regression. Because the model did not condition on the matched risk sets, coefficients for sex and age (the matching variables) should not be interpreted as independent etiologic associations.

**Figure 4:**
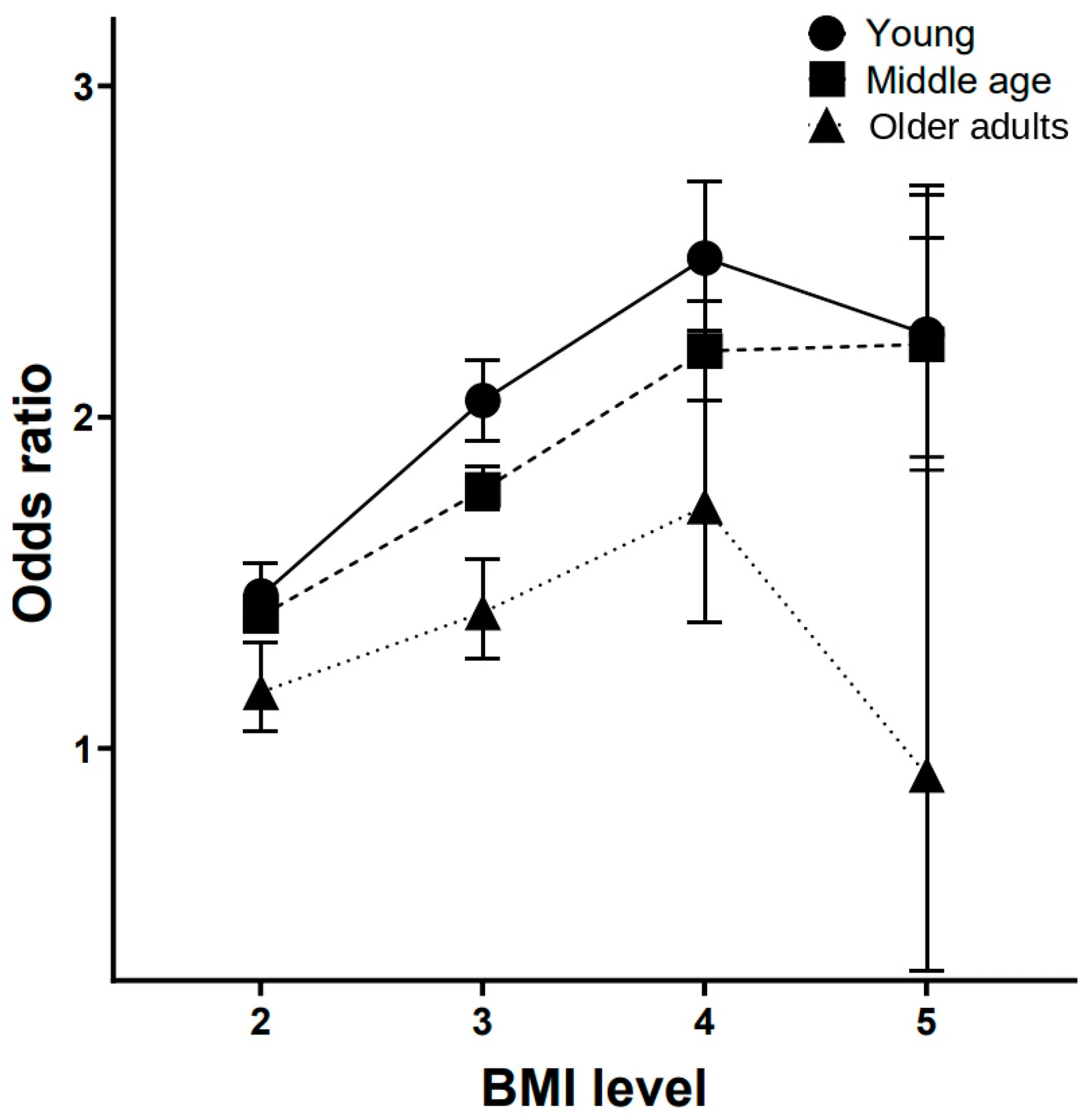
Adjusted odds ratios with 95% confidence intervals for ATR according to BMI level, stratified by age group (young, 23–39 years; middle age, 40–64 years; older adults, ≥65 years). BMI level 1 (<23.0 kg/m^2^) is the reference category; level 2, 23.0–24.9; level 3, 25.0–29.9; level 4, 30.0–34.9; level 5, ≥35.0 kg/m^2^. Estimates are from the unconditional logistic model (Table S3). ATR, Achilles tendon rupture; BMI, body mass index.

Levofloxacin prescriptions were associated with greater odds of ATR (aOR 1.28, 95% CI 1.13–1.44). For each agent, the comparator comprised participants not exposed to that particular agent under the study definition, potentially including users of other fluoroquinolones. Several agents had very few exposed cases and broad confidence intervals, so their non-significant results do not indicate absence of an association. In the overall case–control sample, higher socioeconomic category, certain foot and ankle diagnoses, and higher BMI categories were also associated with greater odds of ATR. BMI 30–34.9 kg/m^2^ was associated with an aOR of 2.20 (95% CI 2.09–2.32) and BMI ≥ 35 kg/m^2^ with an aOR of 2.12 (95% CI 1.87–2.41); the BMI pattern was therefore not strictly monotonic (Figure 4). Achilles tendinitis showed the largest coefficient (aOR 17.58, 95% CI 16.22–19.06).

**Figure 5:**
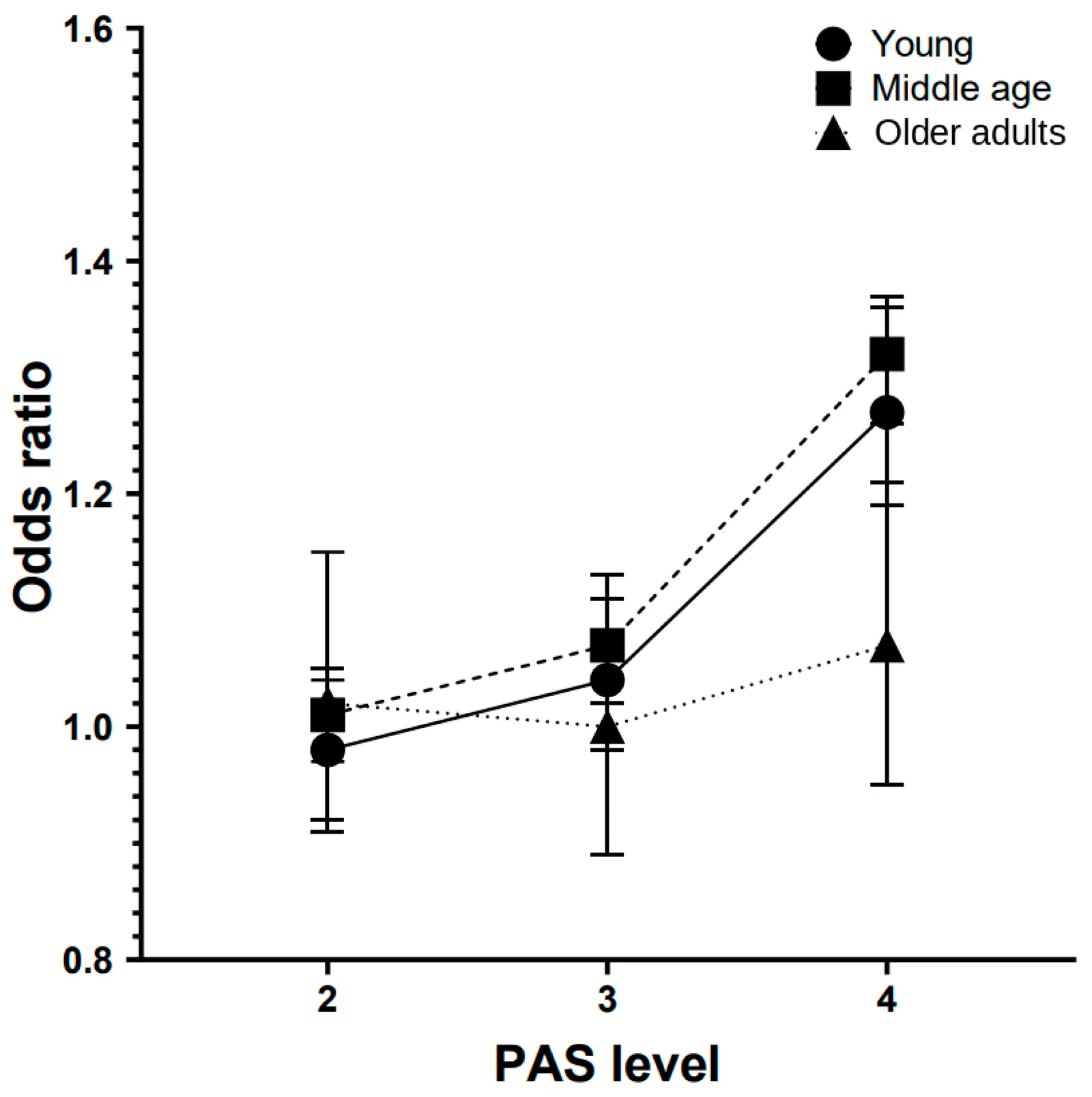
Adjusted odds ratios with 95% confidence intervals for ATR according to physical activity score (PAS) level, stratified by age group (young, 23–39 years; middle age, 40–64 years; older adults, ≥65 years). PAS level 1 (lowest quartile) is the reference category; level 4 is the highest quartile. Estimates are from the unconditional logistic model (Table S3). ATR, Achilles tendon rupture; PAS, physical activity score.

Higher total cholesterol and physical activity categories were associated with greater odds of ATR in the overall sample. PAS level 4 had an aOR of 1.29 (95% CI 1.25–1.34; Figure 5).

### 3.4. Subgroup and Sensitivity Analysis

The age-stratified estimates in Supplementary Table S3 were 1.08 (95% CI 0.82–1.41) for levofloxacin in participants aged 23–39 years, 1.25 (95% CI 1.07–1.46; *p* = 0.005) in those aged 40–64 years, and 1.66 (95% CI 1.23–2.23; *p* < 0.001) in those aged ≥65 years. The direction is consistent with previous reports of higher fluoroquinolone-associated tendon risk in older adults [23,25,26]. Although the point estimates were higher in older groups, formal age-by-treatment interaction was not tested, and these results should not be interpreted as proof of effect modification.

Supplementary Table S4 presents the original alternative adjustment models. Levofloxacin associations remained positive across the reported specifications.

## 4. Discussion

In this nationwide claims-based nested case–control analysis, levofloxacin prescriptions were associated with increased odds of ATR, and the association was more pronounced in middle-aged and older adults. Other agents were infrequently recorded, limiting precision and preventing comparative safety conclusions. BMI, activity, alcohol consumption, socioeconomic category, and comorbidity associations were estimated in the overall sample rather than specifically among fluoroquinolone-exposed participants. The rate analysis also showed an increasing incidence of ATR over time, consistent with the previous analysis of the same database for 2009 to 2017 [21].

The overall incidence of 29.1 per 100,000 persons is comparable to the rates reported from Ontario, Canada (18.0 to 29.3 per 100,000 person-years) [3] and from the previous Korean analysis (20.75 to 26.53 per 100,000 person-years) [21], and higher than the rate estimated from a national emergency department sample in the United States (1.8 to 2.5 per 100,000 person-years) [2]. Direct comparison with clinically confirmed rupture incidence requires caution because the outcome definitions are not equivalent.

### 4.1. Mechanism of ATR and Types of Fluoroquinolones

Fluoroquinolone-associated tendon injury may involve changes in extracellular matrix turnover, oxidative stress, and tendon-cell function, as suggested by experimental studies [27,28]. Published observational studies have reported heterogeneous agent-specific estimates, reflecting differences in populations, prescriptions, analytic approaches, and outcome definitions [12,23]. Fluoroquinolones also differ in pharmacokinetic and pharmacodynamic characteristics [29]. Levofloxacin showed an association in our analysis whereas ofloxacin, its racemate, did not; given the imprecision of the ofloxacin estimate (aOR 0.92, 95% CI 0.73–1.16), this difference does not demonstrate a mechanistic difference or superior safety of other agents.

### 4.2. Factors Associated with ATR in the Overall Sample

Population-level incidence and coefficients estimated in a matched case–control sample address different questions. Sex and age were matching variables, and their unconditional logistic regression coefficients do not estimate their effects and are not interpreted here. In age-stratified analyses, the levofloxacin point estimates increased across age groups, which is consistent with previous reports of older age as a risk factor for fluoroquinolone-associated tendon injury [23,25,26], although a formal interaction analysis was not performed. Previous observational studies have evaluated fluoroquinolone-related tendon events and associated factors, including age, sex, comorbidity, and corticosteroid coexposure [23,25,26,30,31,32,33,34]. In the present study, the associations with BMI, cholesterol, physical activity, alcohol consumption, and foot and ankle comorbidities were estimated in the full sample rather than specifically after fluoroquinolone exposure; whether these factors modify the fluoroquinolone-related association was not tested. Achilles tendinitis and other musculoskeletal diagnoses were established before the outcome index date, not necessarily before antibiotic exposure, so adjustment for tendinitis occurring after treatment may have attenuated the observed drug association.

Metabolic and endocrine conditions may also contribute to Achilles tendon vulnerability independently of antibiotic exposure. Diabetes mellitus, obesity, dyslipidemia, and thyroid dysfunction have been implicated in altered tendon extracellular matrix homeostasis, mechanical properties, and healing capacity [35]. In a multicenter study of 340 patients who underwent surgical repair of Achilles tendon rupture, Oliva et al. found that dysmetabolic disorders were associated with greater odds of postoperative medical complications and a lower likelihood of returning to sports or work [35]. Because that study evaluated patients with an established rupture, its findings do not directly establish the risk of rupture attributable to metabolic disease or an interaction with fluoroquinolone use. Notably, claims-defined diabetes mellitus was not positively associated with ATR in our adjusted model (aOR 0.88, 95% CI 0.84–0.93), which may reflect the limitations of diagnosis-code-based ascertainment, differences in physical activity or healthcare utilization, or residual confounding rather than a protective effect. Our adjusted models included diabetes mellitus, BMI, and total cholesterol; however, these measures may not fully capture glycemic control, lipid abnormalities, endocrine dysfunction, or disease severity. Residual confounding from these incompletely characterized factors therefore remains possible, and the observed association with levofloxacin should be interpreted in the context of the multifactorial etiology of ATR.

### 4.3. Clinical Application

Regulatory agencies already recommend caution regarding systemic fluoroquinolone-associated tendon adverse effects [17,18]. However, the present data do not establish that alcohol abstinence, exercise restriction, or selection of a different fluoroquinolone would reduce the occurrence of fluoroquinolone-associated ATR. Decisions about antibiotic treatment should follow established safety information, appropriate infectious indications, and individual clinical assessment.

The levofloxacin finding should therefore be considered an observational association, not evidence that alternative agents are safer or that prescribing can be individualized using BMI, alcohol, or activity coefficients from this sample.

### 4.4. Limitations

Several limitations should be considered. First, KCD code S86.0 identifies Achilles tendon injury and is not specific to clinically confirmed complete rupture, although it has been used for ATR in previous Korean NHIS studies [21]. The absence of imaging, surgery-based verification, or chart validation may introduce outcome misclassification; its direction is uncertain. Second, the matched risk-set design was analyzed with unconditional logistic regression rather than a matched-set conditional model. Consequently, the effect estimates and standard errors may be affected by incomplete accounting for matching, and sex and age coefficients require particularly cautious interpretation. Third, the exposure definition excluded prescriptions lasting six days or less and included prescriptions up to 180 days before the index date; treatment duration, cumulative dose, recent exposure, and concurrent antibiotic courses were not separately characterized. Fourth, concomitant systemic corticosteroids, clinical indications, infection severity, and healthcare utilization were not fully addressed, leaving potential residual confounding. Fifth, Achilles tendinitis and other foot and ankle diagnoses may have been recorded after exposure; adjusting for potential intermediate variables may bias the estimated association, and the large coefficient for Achilles tendinitis (aOR 17.58) should be read with this in mind. The alternative adjustment models in Table S4 do not replace a matched-set conditional analysis, exposure-window analyses, or exclusion of potential mediators. Sixth, drug-specific comparators were not uniformly fluoroquinolone-unexposed, and sparse exposure in several groups, together with multiple comparisons, limits assessment of between-agent differences. Formal exposure-by-covariate interaction testing was not performed, so patient-characteristic coefficients should not be interpreted as fluoroquinolone-specific susceptibility factors. Finally, this Korean claims-based population may not be generalizable to other settings. Validation of the outcome definition and analyses accounting for matching, temporality, and time-varying exposures are needed before causal or prescribing conclusions are drawn. Future prospective studies may investigate quantitative ultrasound techniques such as shear wave elastography to characterize tendon properties, but these techniques were not evaluated here or established as screening tests for fluoroquinolone-associated injury [36].

## 5. Conclusions

In this nested case–control analysis of Korean NHIS data, levofloxacin exposure was associated with higher odds of ATR (aOR 1.28, 95% CI 1.13–1.44), particularly in older adults. Higher BMI, higher total cholesterol, higher physical activity, heavy alcohol consumption, and several comorbidities were associated with ATR in the overall sample. The limitations of this analysis—a claims-based outcome not restricted to confirmed complete rupture, unconditional analysis of a matched design, exposure misclassification, and residual confounding—preclude causal inference or claims of comparative safety between fluoroquinolones. Further studies with validated outcomes and appropriately matched analyses are required.

## Figures and Tables

**Table 1:** Incidence rate ratio from Poisson regression analysis.

Variables	*n*	Rate (per 10^5^ Person)	IRR (95% CI)	*p*-Value
Female sex	35,827	17.10	0.41 (0.41–0.42)	**<0.001**
Age group				
-young (23–39)	44,085	31.01	reference	-
-middle age (40–64)	68,274	31.51	1.01 (0.995–1.02)	0.224
-older adults (65-)	9802	16.25	0.52 (0.50–0.53)	**<0.001**
Year				
-2009	8584	24.11	reference	**-**
-2010	9069	24.85	1.03 (1.02–1.06)	**<0.001**
-2011	10,019	27.18	1.13 (1.10–1.16)	**<0.001**
-2012	10,270	27.59	1.15 (1.12–1.19)	**<0.001**
-2013	11,094	29.49	1.23 (1.20–1.27)	**<0.001**
-2014	11,152	29.30	1.23 (1.20–1.26)	**<0.001**
-2015	11,965	31.03	1.31 (1.27–1.34)	**<0.001**
-2016	12,367	31.69	1.34 (1.30–1.37)	**<0.001**
-2017	12,260	31.06	1.31 (1.28–1.35)	**<0.001**
-2018	12,381	31.02	1.32 (1.28–1.35)	**<0.001**
-2019	13,000	32.25	1.37 (1.34–1.41)	**<0.001**
Month				
-Jan	7056	1.68	reference	**-**
-Feb	7086	1.69	1.00 (0.97–1.04)	0.801
-Mar	10,293	2.46	1.46 (1.42–1.50)	**<0.001**
-Apr	12,030	2.87	1.70 (1.66–1.76)	**<0.001**
-May	13,673	3.26	1.94 (1.88–1.99)	**<0.001**
-Jun	13,015	3.11	1.84 (1.79–1.90)	**<0.001**
-Jul	12,037	2.87	1.71 (1.66–1.76)	**<0.001**
-Aug	10,807	2.58	1.53 (1.49–1.58)	**<0.001**
-Sep	9641	2.30	1.37 (1.33–1.41)	**<0.001**
-Oct	10,570	2.52	1.50 (1.45–1.54)	**<0.001**
-Nov	8762	2.09	1.24 (1.20–1.28)	**<0.001**
-Dec	7191	1.72	1.02 (0.99–1.05)	0.258

IRRs were calculated with multivariable Poisson regression using sex, age group, year, and month as covariates. ATR was defined by KCD code S86.0. Bold *p*-values indicate statistical significance (*p* < 0.05).

**Table 2:** Characteristics of case and control groups included in logistic regression.

Variables	ATR Group (*n* = 40,240)	Non-ATR Group (*n* = 160,960)	*p*-Value
Female sex, *n* (%)	10,991 (27.3%)	43,964 (27.3%)	1.000
Age group, *n* (%)			0.832
-young (23–39)	11,639 (28.9%)	46,692 (29.0%)	
-middle age (40–64)	25,549 (63.5%)	102,192 (63.5%)	
-older adults (65-)	3052 (7.6%)	12,076 (7.5%)	
Region (Urban, *n* (%)) *	36,388 (90.4%)	147,494 (91.6%)	**<0.001**
Socioeconomic level, *n* (%) **			**<0.001**
-1 (lowest)	5198 (13.6%)	23,169 (14.9%)	
-2	6909 (18.0%)	30,628 (19.6%)	
-3	10,564 (27.5%)	45,820 (29.4%)	
-4 (highest)	15,674 (40.9%)	56,256 (36.1%)	
Comorbidity, *n* (%)			
-Peripheral vascular	2557 (6.4%)	8255 (5.1%)	**<0.001**
-Chronic pulmonary	8491 (21.1%)	30,554 (19.0%)	**<0.001**
-Rheumatologic	1047 (2.6%)	2727 (1.7%)	**<0.001**
-Mild liver	6084 (15.1%)	21,329 (13.3%)	**<0.001**
-DM	3605 (9.0%)	13,736 (8.5%)	**0.007**
-Complication DM	1066 (2.6%)	4042 (2.5%)	0.120
-Renal	72 (0.2%)	221 (0.1%)	0.059
-Severe liver	59 (0.1%)	287 (0.2%)	0.192
-PTTD	40 (0.1%)	47 (0.0%)	**<0.001**
-Pes cavus	15 (0.0%)	10 (0.0%)	**<0.001**
-Metatarsalgia	104 (0.3%)	169 (0.1%)	**<0.001**
-Hemodialysis	18 (0.0%)	82 (0.1%)	0.708
-Plantar fasciitis	1032 (2.6%)	1158 (0.7%)	**<0.001**
-Achilles tendinitis	3574 (8.9%)	803 (0.5%)	**<0.001**
CCI level, *n* (%)			**<0.001**
-0	21,017 (52.2%)	90,812 (56.4%)	
-1	10,846 (27.0%)	40,938 (25.4%)	
-2	4813 (12.0%)	17,158 (10.7%)	
-3	3564 (8.9%)	12,052 (7.5%)	
BMI level, *n* (%)			**<0.001**
-1	10,184 (25.3%)	59,345 (36.9%)	
-2	9714 (24.1%)	40,186 (25.0%)	
-3	16,953 (42.1%)	53,028 (32.9%)	
-4	2994 (7.4%)	7413 (4.6%)	
-5	395 (1.0%)	988 (0.6%)	
Proteinuria level			0.488
-1	38,228 (95.0%)	153,137 (95.1%)	
-2	1103 (2.7%)	4261 (2.6%)	
-3	909 (2.3%)	3562 (2.2%)	
Hemoglobin, g/dL	14.7 ± 1.5	14.6 ± 1.5	**<0.001**
Total cholesterol level			**<0.001**
-1	5159 (12.8%)	24,654 (15.3%)	
-2	6952 (17.3%)	29,897 (18.6%)	
-3	8802 (21.9%)	36,363 (22.6%)	
-4	13,591 (33.8%)	51,089 (31.7%)	
-5	5736 (14.3%)	18,957 (11.8%)	
AST level			**<0.001**
-1	31,582 (78.5%)	130,606 (81.1%)	
-2	5072 (12.6%)	17,899 (11.1%)	
-3	3363 (8.4%)	11,517 (7.2%)	
-4	223 (0.6%)	938 (0.6%)	
ALT level			**<0.001**
-1	28,233 (70.2%)	120,240 (74.7%)	
-2	4667 (11.6%)	16,251 (10.1%)	
-3	6571 (16.3%)	22,139 (13.8%)	
-4	769 (1.9%)	2330 (1.4%)	
Creatinine, mg/dL	1.0 ± 0.8	0.9 ± 0.7	**0.012**
Alcohol consumption			**<0.001**
-non or mild	37,433 (93.0%)	150,652 (93.6%)	
-heavy	2807 (7.0%)	10,308 (6.4%)	
PAS level			**<0.001**
-1	7992 (19.9%)	35,170 (21.9%)	
-2	10,517 (26.1%)	45,866 (28.5%)	
-3	10,301 (25.6%)	42,173 (26.2%)	
-4	11,430 (28.4%)	37,751 (23.5%)	
Drug intake			
-Ciprofloxacin	308 (0.8%)	1074 (0.7%)	**0.036**
-Levofloxacin	412 (1.0%)	1198 (0.7%)	**<0.001**
-Fleroxacin	1 (0.0%)	1 (0.0%)	0.860
-Lomefloxacin	13 (0.0%)	39 (0.0%)	0.467
-Norfloxacin	1 (0.0%)	4 (0.0%)	1.000
-Ofloxacin	102 (0.3%)	398 (0.2%)	0.867
-Moxifloxacin	14 (0.0%)	79 (0.0%)	0.288

BMI categories (kg/m^2^): level 1, <23; level 2, 23–24.9; level 3, 25–29.9; level 4, 30–34.9; level 5, ≥35. CCI categories: 0, 1, 2, and ≥3. Proteinuria categories: negative; trace or 1+; ≥2+. PAS categories represent four sample-based quantile groups (see Table S1). CCI, Charlson comorbidity index; BMI, body mass index; AST, aspartate aminotransferase; ALT, alanine aminotransferase; PAS, physical activity scale. Socioeconomic level was categorized into four levels by insurance premium quantiles. CCI was categorized into four levels. BMI was calculated by weight of body (kg) divided by height (m) squared. BMI level was set referring to the guidelines of the WHO. Proteinuria was measured using the urine dipstick test negative, trace, 1+, 2+, 3+, or 4+; proteinuria was categorized into three levels. Total cholesterol was categorized into five levels. AST and ALT were categorized into four levels. Alcohol consumption was categorized into two levels. Physical activity score (PAS) was calculated depending on the three questionnaires of health examination. The question regarded the number of PA sessions in a week and PA was classified into three classes of physical intensity (light, moderate, vigorous). PAS was based on the metabolic equivalent of tasks (METs). PAS was categorized into four levels and divided for quantiles. The results are given with mean and standard deviation or number of patients and percentage. * Region consisted of urban or rural. ** Socioeconomic level had different numbers of participants. Socioeconomic level data were missing for some participants (cases, *n* = 1895; controls, *n* = 5087); percentages were calculated among participants with available data. Socioeconomic level was based on health insurance premiums, which may be missing when personal information such as occupation is not disclosed. Bold *p*-values indicate statistical significance (*p* < 0.05).

**Table 3:** Results of multiple logistic regression analysis.

Variables	Crude OR	*p*-Value	Adjusted OR	*p*-Value
Drug intake				
-Ciprofloxacin	1.15 (1.01–1.30)	**0.03**	1.05 (0.91–1.20)	0.512
-Levofloxacin	1.38 (1.23–1.54)	**<0.001**	1.28 (1.13–1.44)	**<0.001**
-Fleroxacin	4.00 (0.25–63.96)	0.327	5.22 (0.32–85.22)	0.246
-Lomefloxacin	1.33 (0.71–2.50)	0.369	1.28 (0.66–2.47)	0.462
-Norfloxacin	1.00 (0.11–8.95)	1.000	0.99 (0.11–8.94)	0.993
-Ofloxacin	1.03 (0.82–1.27)	0.823	0.92 (0.73–1.16)	0.465
-Moxifloxacin	0.71 (0.40–1.25)	0.235	0.67 (0.36–1.23)	0.195
Female sex, *n* (%)	1.00 (0.98–1.02)	1.000	1.23 (1.19–1.28)	**<0.001**
Age group, years				
-young (23–39)	reference	-	reference	-
-middle age (40–64)	1.00 (0.98–1.03)	0.813	0.91 (0.88–0.93)	**<0.001**
-older adults (65-)	1.01 (0.97–1.06)	0.601	0.86 (0.81–0.90)	**<0.001**
Region (Urban)	0.86 (0.83–0.90)	**<0.001**	0.86 (0.83–0.90)	**<0.001**
Socioeconomic level				
-1 (lowest)	reference	-	reference	-
-2	1.01 (0.97–1.05)	0.788	1.02 (0.98–1.07)	0.247
-3	1.03 (0.99–1.07)	0.146	1.03 (0.99–1.08)	0.090
-4 (highest)	1.24 (1.20–1.29)	**<0.001**	1.26 (1.21–1.31)	**<0.001**
Comorbidity				
-Peripheral vascular	1.26 (1.20–1.31)	**<0.001**	1.07 (1.01–1.14)	**0.014**
-Chronic pulmonary	1.14 (1.11–1.17)	**<0.001**	1.02 (0.98–1.06)	0.297
-Rheumatologic	1.55 (1.44–1.67)	**<0.001**	1.26 (1.16–1.37)	**<0.001**
-Mild liver	1.17 (1.13–1.20)	**<0.001**	1.01 (0.96–1.05)	0.747
-DM	1.05 (1.01–1.10)	**<0.001**	0.88 (0.84–0.93)	**<0.001**
-Complication DM	1.06 (0.99–1.13)	0.116	0.96 (0.88–1.04)	0.269
-Renal	1.30 (1.00–1.70)	0.051	1.18 (0.87–1.59)	0.291
-Severe liver	0.82 (0.62–1.09)	0.171	0.71 (0.52–0.96)	**0.027**
-PTTD	3.41 (2.23–5.19)	**<0.001**	3.04 (1.91–4.83)	**<0.001**
-Pes cavus	6.00 (2.70–13.36)	**<0.001**	2.64 (1.05–6.65)	**0.040**
-Metatarsalgia	2.47 (1.93–3.15)	**<0.001**	1.35 (1.01–1.81)	**0.042**
-Hemodialysis	0.88 (0.53–1.46)	0.617	0.83 (0.47–1.48)	0.528
-Plantar fasciitis	3.63 (3.34–3.95)	**<0.001**	2.03 (1.84–2.24)	**<0.001**
-Achilles tendinitis	19.44 (17.99–21.01)	**<0.001**	17.58 (16.22–19.06)	**<0.001**
CCI level				
-0	reference	–	reference	-
-1	1.14 (1.12–1.17)	**<0.001**	1.09 (1.05–1.13)	**<0.001**
-2	1.21 (1.17–1.26)	**<0.001**	1.12 (1.06–1.18)	**<0.001**
-3	1.28 (1.23–1.33)	**<0.001**	1.17 (1.07–1.27)	**<0.001**
BMI level				
-1	reference	–	reference	-
-2	1.41 (1.37–1.45)	**<0.001**	1.38 (1.33–1.42)	**<0.001**
-3	1.86 (1.81–1.91)	**<0.001**	1.78 (1.73–1.84)	**<0.001**
-4	2.35 (2.24–2.47)	**<0.001**	2.20 (2.09–2.32)	**<0.001**
-5	2.33 (2.07–2.62)	**<0.001**	2.12 (1.87–2.41)	**<0.001**
Proteinuria level				
-1	reference	–	reference	-
-2	1.04 (0.97–1.11)	0.289	0.99 (0.92–1.06)	0.824
-3	1.02 (0.97–1.11)	0.558	0.95 (0.87–1.02)	0.172
Hemoglobin, g/dL	1.03 (1.03–1.04)	**<0.001**	1.02 (1.01–1.03)	**0.003**
Total cholesterol level				
-1	reference	–	reference	-
-2	1.11 (1.07–1.16)	**<0.001**	1.10 (1.05–1.15)	**<0.001**
-3	1.16 (1.11–1.20)	**<0.001**	1.10 (1.06–1.15)	**<0.001**
-4	1.27 (1.23–1.32)	**<0.001**	1.18 (1.13–1.22)	**<0.001**
-5	1.45 (1.39–1.51)	**<0.001**	1.25 (1.19–1.31)	**<0.001**
AST level				
-1	reference	–	reference	-
-2	1.17 (1.13–1.21)	**<0.001**	1.03 (0.99–1.07)	0.119
-3	1.21 (1.16–1.26)	**<0.001**	1.03 (0.97–1.08)	0.317
-4	0.98 (0.85–1.14)	0.820	0.87 (0.72–1.05)	0.148
ALT level				
-1	reference	–	reference	-
-2	1.22 (1.18–1.27)	**<0.001**	1.03 (0.99–1.07)	0.160
-3	1.26 (1.23–1.30)	**<0.001**	1.02 (0.98–1.06)	0.425
-4	1.41 (1.29–1.53)	**<0.001**	1.06 (0.95–1.19)	0.265
Creatinine, mg/dL	1.03 (1.01–1.04)	**<0.001**	1.02 (1.01–1.04)	**0.002**
Alcohol consumption				
-non or mild	reference	–	reference	-
-heavy	1.10 (1.05–1.14)	**<0.001**	1.11 (1.06–1.16)	**<0.001**
PAS level				
-1	reference	–	reference	-
-2	1.01 (0.98–1.04)	0.583	1.00 (0.97–1.04)	0.878
-3	1.07 (1.04–1.11)	**<0.001**	1.06 (1.02–1.10)	**<0.001**
-4	1.33 (1.29–1.38)	**<0.001**	1.29 (1.25–1.34)	**<0.001**

Odds ratios are from the original unconditional logistic model; matching on sex, age, and examination year was not accounted for, because a conditional risk-set analysis was not performed. Sex and age coefficients should not be interpreted as independent etiologic effects. Agent-specific reference groups comprise individuals not exposed to the indicated agent and may include exposure to other fluoroquinolones. Exposed case and control counts are shown in Table 2. Bold *p*-values indicate statistical significance (*p* < 0.05).

## Data Availability

The data that support the findings of this study are available from the Korean National Health Insurance Service (NHIS), but restrictions apply to the availability of these data, which were used under license for the current study and are therefore not publicly available. Data may be available from the corresponding author upon reasonable request and with permission from the NHIS.

## Supplementary Materials

The following supporting information can be downloaded at https://www.mdpi.com/article/10.3390/jcm15218404/s1. Table S1: Definition of covariates; Table S2: Comorbidity; Table S3: Sub-analysis of logistic regression analysis (age-stratified); Table S4: Sensitivity analysis.
