# Supplementary material for: Fluoroquinolone Exposure and Other Factors Associated with Achilles Tendon Rupture: A Nationwide Nested Case–Control Study
Source: J Clin Med. 2026 Oct 28;15(21):8404. doi: 10.3390/jcm15218404 (PMC13763662; doi:10.3390/jcm15218404)
Supplement: Supplementary file 1 [file jcm-15-08404-s001.zip › jcm-4612881-supplementary.pdf]

Supplementary Table S1. Definition of covariates

Variable	Classification
Age group	Young (23~39), Middle (40~64), Older adults (over 65)
Residence area	Urban, Rural
Socioeconomic level	1, 2, 3, 4
CCI level	0, 1, 2, 3 (over 3)
BMI level	1(under 23), 2(23~24.9), 3(25~29.9), 4(30~34.9), 5(over 35)
Proteinuria level	1(negative), 2(trace or 1+), 3(2+ or over)
Total cholesterol level(mg/dL)	1(under 159.9), 2(160 ~ 179.9), 3(180 ~ 199.9), 4(200 ~ 239.9), 5(over 240)
AST for male(U/L)	1(under 32.9), 2(33 ~ 39.9), 3(40 ~ 99.9), 4(over 100)
AST for female(U/L)	1(under 24.9), 2(25 ~ 39.9), 3(40 ~ 99.9), 4(over 100)
ALT for male(U/L)	1(under 32.9), 2(33 ~ 39.9), 3(40 ~ 99.9), 4(over 100)
ALT for female(U/L)	1(under 24.9), 2(25 ~ 39.9), 3(40 ~ 99.9), 4(over 100)
Alcohol consumption	non or mild, heavy
PAS	1, 2, 3, 4

CCI, Charlson comorbidity index; BMI, body mass index; AST, Aspartate aminotransferase; ALT, Alanine Aminotransferase; PAS, Physical activity scale

Socioeconomic level was categorized to four levels by insurance premium quantiles.

CCI level was categorized into four levels by CCI (Charlson, Pompei, Ales, & MacKenzie, 1987).

BMI was calculated that weight of body (kg) was divided by height (m) squared. BMI level was set referring to the Asia-Pacific regional guidelines of the WHO.

Proteinuria was measured using the urine dipstick test negative, trace, 1+, 2+, 3+, or 4+). Proteinuria level was categorized into three levels.(S. K. Park et al., 2021).

Total cholesterol level was categorized into five levels (Kitahara et al., 2011).

AST, ALT were categorized into four levels (Oh, Jang, & Song, 2021).

Alcohol consumption was categorized into two level(Lee et al., 2019).

Physical activity score (PAS) was calculated depending on the three questionnaire of health examination. The question was how many times PA in a week and PA was classified three class by physical intensity (light, moderate, vigorous). PAS was based from metabolic equivalent of tasks (METs) (Kwon et al., 2021). PAS was categorized into four level and divided for quantiles.

Supplementary Table S2. Comorbidity

Variable	KCD code
Peripheral vascular disease	I70.x, I71.x, I73.1, I73.8, I73.9, I77.1, I79.0, I79.2, K55.1, K55.8, K55.9, Z95.8, Z95.9
Chronic pulmonary disease	I27.8, I27.9, J40.x-J47.x, J60.x-J67.x, J68.4, J70.1, J70.3
Rheumatologic disease	M05.x, M06.x, M31.5, M32.x-M34.x, M35.1, M35.3, M36.0
Mild liver disease	B18.x, K70.0-K70.3, K70.9, K71.3-K71.5, K71.7, K73.x, K74.x, K76.0, K76.2-K76.4, K76.8, K76.9, Z94.4
Diabetes mellitus (DM)	E10.0, E10.1, E10.6, E10.8, E10.9, E11.0, E11.1, E11.6, E11.8, E11.9, E12.0, E12.1, E12.6, E12.8, E12.9, E13.0, E13.1, E13.6, E13.8, E13.9, E14.0, E14.1, E14.6, E14.8, E14.9
Complication DM	E10.2-E10.5, E10.7, E11.2-E11.5, E11.7, E12.2-E12.5, E12.7, E13.2-E13.5, E13.7, E14.2-E14.5, E14.7
Renal disease	I12.0, I13.1, N03.2-N03.7, N05.2-N05.7, N18.x, N19.x, N25.0, Z49.0-Z49.2, Z94.0, Z99.2
Severe liver disease	I85.0, I85.9, I86.4, I98.2, K70.4, K71.1, K72.1, K72.9, K76.5, K76.6, K76.7
PTTD	M768
Pes cavus deformity	Q667
Metatarsalgia	M774
Plantar fasciitis	M722
Achilles tendinitis	M766

Peripheral vascular, Chronic pulmonary, Rheumatologic, Mild liver, DM, Complication DM, Renal, Severe liver were referenced CCI.
Hemodialysis(O7020) was searched in procedure code.
PTTD, posterior tibial tendon dysfunction

Supplementary Table S3. Sub-analysis of logistic regression analysis

Age group	**Young**		**Middle age**		**Older adults**	
	OR(95% CI)	p-value	OR(95% CI)	p-value	OR(95% CI)	p-value
Ciprofloxacin	1.24(0.94~1.62)	0.127	0.98(0.82~1.17)	0.801	1.09(0.76~1.56)	0.634
Levofloxacin	1.08(0.82~1.41)	0.603	1.25(1.07~1.46)	**0.005**	1.66(1.23~2.23)	**<0.001**
Ofloxacin	0.92(0.53~1.62)	0.780	0.91(0.69~1.21)	0.520	0.99(0.54~1.81)	0.966
BMI level 1	reference	-	reference	-	reference	-
BMI level 2	1.46(1.37~1.56)	**<0.001**	1.40(1.35~1.46)	**<0.001**	1.17(1.05~1.32)	**0.007**
BMI level 3	2.05(1.93~2.17)	**<0.001**	1.78(1.72~1.85)	**<0.001**	1.41(1.27~1.57)	**<0.001**
BMI level 4	2.48(2.26~2.71)	**<0.001**	2.20(2.05~2.35)	**<0.001**	1.73(1.38~2.17)	**<0.001**
BMI level 5	2.25(1.88~2.70)	**<0.001**	2.22(1.84~2.67)	**<0.001**	0.92(0.33~2.54)	0.871
PAS level 1	reference	-	reference	-	reference	-
PAS level 2	0.98(0.92~1.04)	0.488	1.01(0.97~1.05)	0.701	1.02(0.91~1.15)	0.714
PAS level 3	1.04(0.98~1.11)	0.212	1.07(1.02~1.11)	**0.004**	1.00(0.89~1.13)	0.952
PAS level 4	1.27(1.19~1.36)	**<0.001**	1.32(1.26~1.37)	**<0.001**	1.07(0.95~1.21)	0.282

Model: Sex + Age + Region + Socioeconomic level + Comorbidity + CCI level + BMI level + Proteinuria level + Hemoglobin +
Total cholesterol level + AST level + ALT level + Creatinine + Alcohol consumption +
PAS level + Ciprofloxacin + Levofloxacin + Ofloxacin

Ciprofloxacin, Levofloxacin, Ofloxacin used as sub-analysis outcome. Because other drugs were excluded due to lack of participants. BMI level1 and PAS level1 were abbreviated as reference.

Supplementary Table S4. Sensitivity analysis

	Ciprofloxacin		Levofloxacin		Ofloxacin	
Model	OR(95% CI)	p-value	OR(95% CI)	p-value	OR(95% CI)	p-value
Model1	1.11(0.98~1.27)	0.106	1.37(1.22~1.54)	**<0.001**	1.01(0.81~1.26)	0.956
Model2	1.04(0.91~1.19)	0.578	1.24(1.10~1.40)	**<0.001**	0.93(0.74~1.17)	0.531
Model3	1.06(0.93~1.22)	0.380	1.28(1.13~1.44)	**<0.001**	0.92(0.73~1.16)	0.496

Model1: Sex + Age group + Region + Socioeconomic level+ Ciprofloxacin + Levofloxacin + Ofloxacin + Fleroxacin + Lomefloxacin + Norfloxacin + Moxifloxacin

Model2: Sex + Age group + Region + Socioeconomic level + Comorbidity + CCI level + BMI level + Proteinuria level + Hemoglobin + Total cholesterol level + AST level + ALT level + Creatinine + Alcohol consumption + PAS level + Ciprofloxacin + Levofloxacin + Ofloxacin

Model3: Sex + Age group + Region + Socioeconomic level + Comorbidity + CCI level + BMI level + Proteinuria level + Hemoglobin + Total cholesterol level + AST level + ALT level + Creatinine + Alcohol consumption + PAS level + Each fluoroquinolone
